# B cells modulate mouse allergen-specific T cells in nonallergic laboratory animal-care workers

**DOI:** 10.1172/jci.insight.145199

**Published:** 2021-02-22

**Authors:** Esther Dawen Yu, Luise Westernberg, Alba Grifoni, April Frazier, Aaron Sutherland, Eric Wang, Bjoern Peters, Ricardo da Silva Antunes, Alessandro Sette

**Affiliations:** 1 La Jolla Institute for Immunology, La Jolla, California, USA.; 2 Department of Medicine, University of California, San Diego, La Jolla, California, USA.

**Keywords:** Immunology, Allergy, Antigen, T cells

## Abstract

Understanding the mechanisms of allergen-specific immune modulation in nonallergic individuals is key to recapitulate immune tolerance and to develop novel allergy treatments. Herein, we characterized mouse-specific T cell responses in nonallergic laboratory animal-care workers before and after reexposure to mice. PBMCs were collected and stimulated with developed peptide pools identified from high-molecular-weight fractions of mouse allergen extracts. Sizable CD4 T cell responses were noted and were temporarily decreased in most subjects upon reexposure, with the magnitude of decrease positively correlated with time of reexposure but not the duration of the break. Interestingly, the suppression was specific to mouse allergens without affecting responses of bystander antigens. Further, PBMC fractioning studies illustrated that the modulation is unlikely from T cells, while B cell depletion and exchange reversed the suppression of responses, suggesting that B cells may be the key modulators. Increased levels of regulatory cytokines (IL-10 and TGF-β1) in the cell culture supernatant and plasma mouse-specific IgG4 were also observed after reexposure, consistent with B cell–mediated modulation mechanisms. Overall, these results suggest that nonallergic status is achieved by an active, time-related, allergen-specific, B cell-dependent regulatory process upon reexposure, the mechanisms of which should be detailed by further molecular studies.

## Introduction

Allergy is an emerging healthcare challenge in Western countries. It appears to be associated with the modern lifestyle, including lower exposure to microbial and parasitic infections due to improved hygiene and antibiotic use (1). Animal allergies are prevalent in inner city households with pets and animal-care workers (2). Among laboratory animals, mouse allergy is the most common occupation-related allergic disease, as rodents are the most frequently used in research. The main sources of allergens are urine, saliva, and dander. Once an individual is sensitized, symptoms may develop within minutes of contact. Studies reported that 10%–44% of laboratory workers developed allergic symptoms from 30 days to years after exposure to animals (3). About 40% had atopic dermatitis and urticaria; up to 80% had allergic rhinoconjunctivitis with rhinorrhea, sneezing, nasal congestion, and itchy, watery eyes; and up to 22% of individuals reported severe respiratory symptoms, indicating asthma exacerbation, i.e., cough, shortness of breath, and wheeze (4). Rarely, life-threatening allergic reactions, such as anaphylaxis with respiratory and circulatory failure could happen following an animal bite or contaminated needlestick. The conventional treatments for allergy consist of allergen avoidance, symptom-relieving pharmacotherapy, and patient education; these treatments are safe and relatively effective but do not change the chronic course of the disease.

The two major therapeutic strategies currently under development are allergen immunotherapy (AIT), which aims to induce protective immune tolerance, and biological immune response modifiers, which target pathological immune responses. While AIT is the only disease-modifying treatment for allergy so far, there are concerns regarding its limited efficacy, side effects, uncertainty of developing permanent tolerance, high cost, and low patient adherence due to the need for long-term treatment (5, 6).

The mechanisms of immune tolerance have been intensively studied. Although it is evident that IFN-γ−producing Th1 cells and IL-10–secreting Tregs play an important role in developing immune tolerance in nonallergic individuals (7), details of this regulatory process have not been fully elucidated. Studies on human high-dose allergen exposure models have demonstrated changes in the profiles of allergen-specific memory T and B cell responses. These result in the production of cytokines and antibodies that skew the immune responses toward a noninflammatory direction, leading to reduction of activation, tissue migration, and degranulation of mast cells, basophils, and eosinophils (8). A recent study on Timothy grass pollen allergen reported seasonal allergen-specific immune modulation in nonallergic individuals, including downregulation of T cell reactivity and consequently reduced IL-5 and increased IFN-γ production during pollen seasons. It was proposed that immune tolerance in exposed nonallergic (ENA) individuals is not an outcome from absence of allergic reactions, but is instead due to an active allergen-specific regulatory process (9).

Laboratory animal-care workers are the ideal cohort to study the immune modulation in allergen tolerance, as most of them do not become sensitized to mouse allergens, despite heavy exposure, and it is relatively easy to monitor their allergen exposure. However, little is known about the mouse allergens so far (10). Mus m 1, a major urinary protein, is the only allergen listed in the International Union of Immunological Societies database (11). Our group previously reported an innovative immunoproteomic approach that revealed the complexity of mouse allergens recognized by human T cells. A total of 106 dominant T cell epitopes from 35 different protein targets were identified from the high-molecular-weight portion of the mouse allergen (12), and an additional 50 epitopes were identified from low-molecular-weight oligopeptides of mouse urine allergens (13). These tools make it possible to study the immune modulation in mouse allergy. Moreover, the protein targets of ENA animal-care workers share high similarity to the those who are allergic to mice. Importantly, these subjects have lower but still sizable T cell reactivity after reexposure (ARE) of mouse allergens. However, compared with allergic subjects where the responses are polarized toward high IL-5 and low IFN-γ production, ENA subjects showed no polarization (14). The cause of the lack of polarization and the regulatory mechanisms in ENA subjects ARE to allergens are currently unknown.

We hypothesize that allergen-specific immune modulation reported in nonallergic individuals in the seasonal pollen allergy study (9) is similar to that in mouse ENA animal-care workers. Using this laboratory mouse allergy model and a newly developed peptide pool of mouse allergens, we studied CD4 T cell responses with the aim to characterize this regulatory process and explore its underlying mechanisms.

## Results

### Establishment of a longitudinal study on ENA workers.

**Figure 1:**
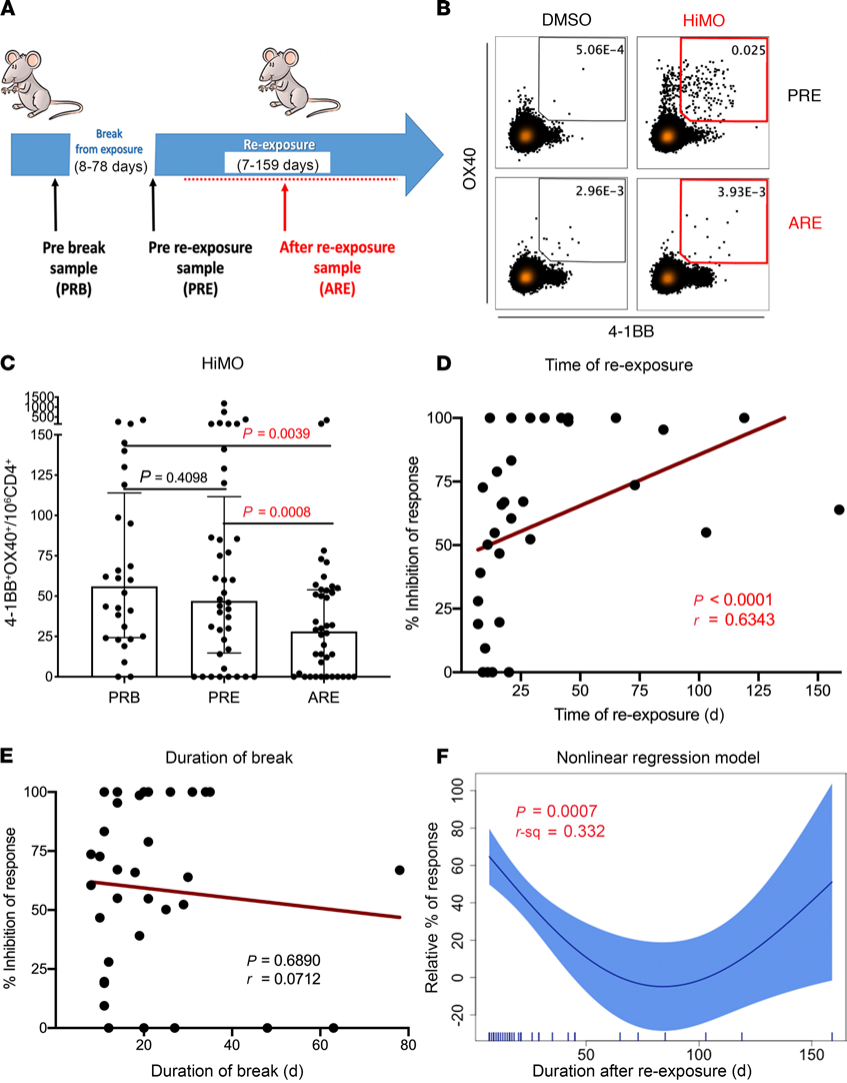
Longitudinal study on exposed nonallergic mouse laboratory workers. (**A**) Diagram illustrating the study design of the mouse longitudinal study. Blood samples were collected at 3 time points: pre-break (PRB), after break but pre-reexposure (PRE), and after reexposure (ARE) to mouse. (**B** and **C**) Decrease in CD4 T cell responses in nonallergic subjects after reexposure to mouse. Differences in T cell reactivity of 3 groups were detected using AIM assay (**C**, *n* = 36). AIM^+^ signals (4-1BB^+^OX40^+^) are represented by numbers per million of CD4 T cells. Data are plotted as median with interquartile range. Statistical analysis was performed by Wilcoxon’s test for paired comparison, with Bonferroni correction for multiple comparison. Time relationship of magnitude of T cell response inhibition was investigated with time of reexposure (**D**) and duration of break (**E**) using Spearman’s correlation test (*n* = 36). Percentage of inhibition = (1 – [ARE response]/[PRE response ]) × 100. (**F**) Plot of a nonlinear regression model based on cross-sectional data (*n* = 36) showing a trend of dynamic changes of CD4 T cell responses ARE. Relative percentage of response = (ARE response/PRE response) × 100. Nonlinear generalized additive model was performed using the mgcv package for R.

To investigate additional differences in immune profiles between ENA and allergic individuals, and whether the nonallergic status is a passive consequence or active response to mouse allergens, a cohort of vivarium and research laboratory ENA subjects regularly working in contact with mice was followed longitudinally. We introduced the breaks of exposure method to study how their T cell responses change after breaks and reexposure to mice. Peripheral blood samples were collected while subjects were continuously exposed (pre-break [PRB]), after break but before reexposure (pre-reexposure [PRE]), and ARE (Figure 1A); break duration ranges were from 8 to 78 days and reexposure time ranges were from 7 to 159 days. Participants had no clinical symptom of mouse allergy and negative laboratory evidence of sensitization, as defined by serum mouse-specific IgE levels of less than 0.10 kU_A_/L. All samples were consistently IgE negative throughout the longitudinal study. The median duration of occupational exposure to mice was 2.4 hours per day, with a range of 0.4 to 8.0 hours per day. The cohort included 20 White, 10 Hispanic or Latino, 4 Asian, and 2 African American subjects, with a median age of 28.7 ± 5.7 years and a male-to-female ratio of 5:7. Demographic and clinical information are summarized in Table 1.

### Decrease of cytokine secretion and T cell responses in ENA subjects upon reexposure to mouse allergens was observed.

In pilot experiments, it was noted that ENA subjects had a decrease in responses to high-molecular-weight fractions (HiMO) of mouse allergen extracts after being reexposed to mouse allergens, as observed in a 2-week restimulation analysis of PBMCs by enzyme-linked immunospot (ELISPOT) assay measuring IFN-γ and IL-5. A total of 6 of 8 (75%) subjects showed reduced secretion of cytokines ARE (Supplemental Figure 1A; supplemental material available online with this article; https://doi.org/10.1172/jci.insight.145199DS1).

To confirm this observation in a larger group of subjects, and to exclude possible artifact introduced by in vitro culture, we used the activation-induced marker (AIM) assay. We and others previously demonstrated that allergen-specific T cells can be detectable ex vivo using this assay in combination with pools of T cell epitopes (12–14). This technique uses the upregulation of the activation markers 4-1BB (CD137) and OX40 (CD134) as a read-out for T cell reactivity and has been applied in previous studies (12–14) to quantify mouse allergen-specific T cells after HiMO peptide stimulation. Representative plots of the mouse allergen-specific T cell responses before reexposure and ARE, as measured by the AIM assay, which detects the fraction of activated CD4 T cells in response to antigen-specific stimulation by measuring the 4-1BB (CD137) and OX40 (CD134) cell markers are shown in Figure 1B. PBMCs from 25 of 36 (69.4%) (Supplemental Figure 1B) ENA subjects had significantly lower responses to the HiMO allergen in the ARE group compared with either the PRB (*P* = 0.0039) or PRE (*P* = 0.0008) groups. No difference in responses was observed between the PRB and PRE samples (*P* = 0.4098) (Figure 1C).

### Magnitude of decrease in T cell responses was positively correlated with time ARE but not duration of break.

Using PBMC samples with different time intervals from reexposure to mice, we found that the magnitude of decrease in T cell responses was time dependent (R = 0.6343, *P* < 0.0001, Figure 1D); longer intervals from reexposure (ranges from 7 to 159 days) were associated with more pronounced decreases in T cell responses. In addition, we studied the effect of the duration of break (ranges from 8 to 78 days), with no significant association observed with a reduction in proportion of activated CD4 T cells (R = 0.0712, *P* = 0.6890, Figure 1E).

### Subjects with 2 break cycles showed repeated decreases of responses and returned to baseline.

To study the course of change in T cell responses ARE, we fitted the above cross-sectional data (Figure 1D) into a nonlinear regression model (Figure 1F), which showed that the T cell responses decreased with time ARE, with a trough approximately around day 90 ARE, before returning to baseline (approximate *P* = 0.0007, adjusted R^2^ = 0.332). However, the results shown in Figure 1, D–F, should be interpreted with caution, given the fact that very few points are available after 50 days.

To verify this finding, subjects who participated in the first cycle of the longitudinal study were recalled, and a second cycle of longitudinal study was subsequently designed with 5 follow-up visits: PRB, PRE, ARE1 (within 1 month), ARE2 (1–3 months), and ARE3 (3–6 months). Unfortunately, this study was affected by the lockdown for the global coronavirus disease 2019 (COVID-19) pandemic, and only 4 subjects completed the ARE1 visit in the second cycle.

By analyzing those 4 subjects, a general trend was observed that CD4 T cell responses were downregulated temporarily after each time of reexposure in all 4 subjects in both cycles and eventually returned to baseline at some point in the first cycle prior to the second break (Supplemental Figure 2, A–D), which fitted within the confidence interval of the nonlinear model predicted in the first cycle. However, these observations should be interpreted with caution, given the fact that number of observations is limited.

### Decrease in T cell responses ARE is an active process.

**Figure 2:**
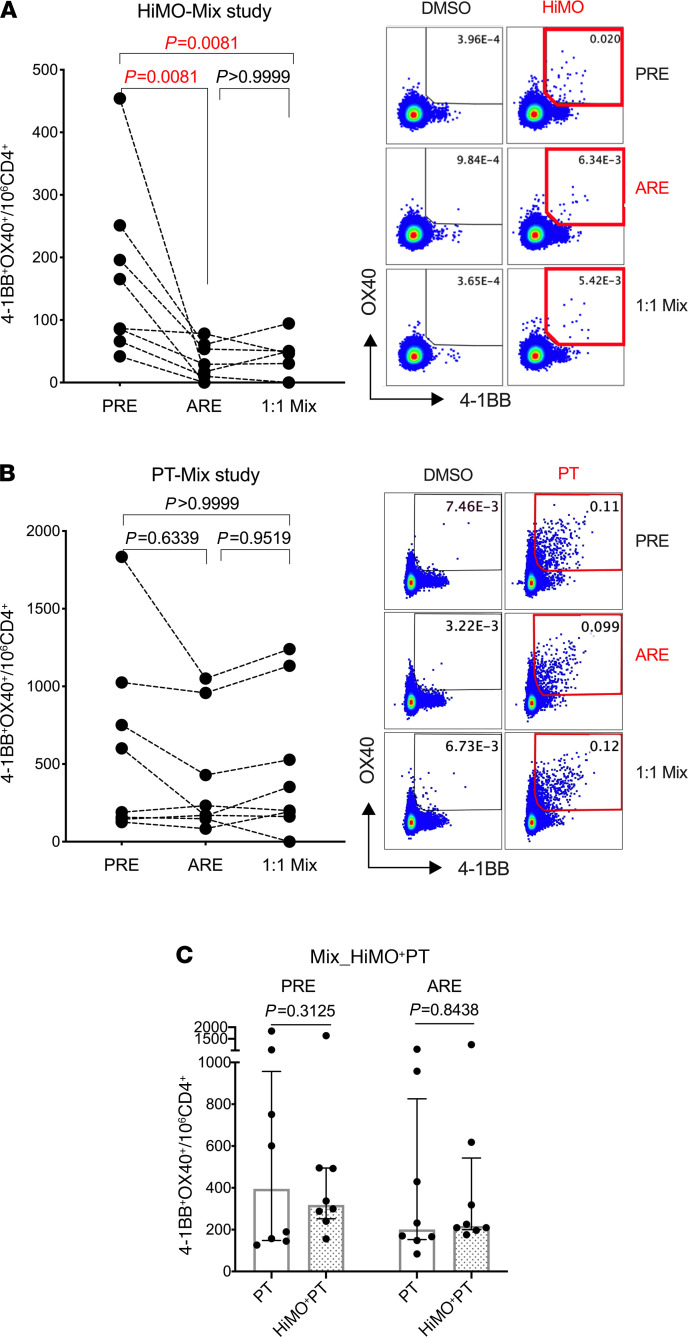
Before/after reexposure cell-mixing study. (**A** and **B**) Coculture of longitudinal samples (pre-break [PRB] and after reexposure [ARE], mixed in a 1:1 ratio, *n* = 8) stimulated by mouse (HiMO) and pertussis (PT) antigens. T cell responses of PRE, ARE, and mixed cultures were detected by AIM assay. AIM^+^ signals (4-1BB^+^ OX40^+^) are represented by numbers per million of CD4 T cells. Comparisons among the 3 groups are shown to the left, and flow cytometry images are shown to the rig^+^ht. Statistical analysis was performed by Friedman’s test corrected for multiple comparison with Dunn’s test. (**C**) Cell cultures were stimulated with PT alone and PT mixed with mouse antigens simultaneously (*n* = 8). T cell responses of PT alone and PT+HiMO were compared with Wilcoxon’s test (2-tailed). Data are plotted as median with interquartile range.

We conducted mixing studies to explore whether the observed decrease in T cell responses was due to active downregulation or a passive consequence from other causes, such as depletion of responding immune cells due to redistribution from blood to tissue. We selected 8 subjects whose responses were observed to be downmodulated in previous experiments, and PBMCs from those 8 subjects at PRE and ARE visits were mixed and cultured at a 1:1 ratio, followed by stimulation with HiMO mouse allergens. T cell responses would still be suppressed in mixed culture if the suppression was due to active downregulation in the ARE samples, in contrast to depletion of immune cells responsive to allergen, which would give rise to an increase in T cell activation from reactive cells in the PRE samples. As shown in Figure 2A, in this new independent assay, we confirmed the originally observed suppressive effect in 8 of 8 subjects (*P* = 0.0081), and we further observed that T cell responses were consistently suppressed in the mixed cultures (*P* = 0.0081), suggesting that the decrease in T cell responses ARE to allergen in the ARE group is an actively suppressive process.

### Downregulation of T cell responses ARE is specific for mouse allergens.

We further evaluated the specificity of the decrease in T cell responses to allergens. Similar mixing studies were conducted using a previously described pool of antigenic peptides in the pertussis (PT) vaccine as representative bystander allergens (15), which did not demonstrate significant decrease of T cell response, as shown in Figure 2B. The antigen-specific nature of the inhibitory effect in response to mouse allergen is further supported by the observations in the 1:1 mixing experiments, with a striking inhibition in the case of mouse responses (*P* = 0.0081) but no effect in the case of PT responses (*P* > 0.9999).

The bystander effect, a nonspecific suppression by mouse allergens of T cell responses to other antigens, was also examined. We stimulated PBMCs with mouse and PT antigens simultaneously and noted that responses to PT were not decreased in either PRE or ARE samples (Figure 2C). In conclusion, downregulation of T cell responses is specific to mouse allergens and not affected bystander antigens.

### Phenotyping PBMCs ARE to mouse allergens.

To gain additional insights into the profile of the cells potentially mediating the downregulation of responses, we performed direct ex vivo immunophenotyping of PBMCs with surface markers for various PBMC subpopulations, including CD8 T cells, monocytes, natural killer cells, natural killer T cells, plasmacytoid dendritic cells, myeloid dendritic cells, CD141^+^ dendritic cells, basophils, Tregs, T follicular helper cells, and follicular Tregs (Supplemental Table 2). No consistent qualitative changes were revealed (Supplemental Figure 3, A–K), apart from significant decreases in B cell (CD14^–^CD3^–^CD19^+^) proportions in ARE compared with PRE (*P* = 0.0110) and PRB (*P* = 0.0417) groups (Supplemental Figure 4, A and B).

In addition, CD4 T cells that expressed common memory cell markers (CCR7 and CD45RA) were quantified and showed a reduction in number of mouse antigen-specific (4-1BB^+^OX40^+^) CD4 effector memory cells (Temra CD45RA^+^CCR7^–^) (*P* = 0.0049) but not total CD4 effector cells (Supplemental Figure 4, C and D). These results were observed in 9 of 16 subjects comparing the ARE and PRB groups (*P* = 0.0049) (8 subjects did not have PRB samples available for testing), and they were consistent with the antigen specificity of the effects as described above.

### Allergen-specific immune modulation is mediated by antigen-presenting cells but not T cells.

**Figure 3:**
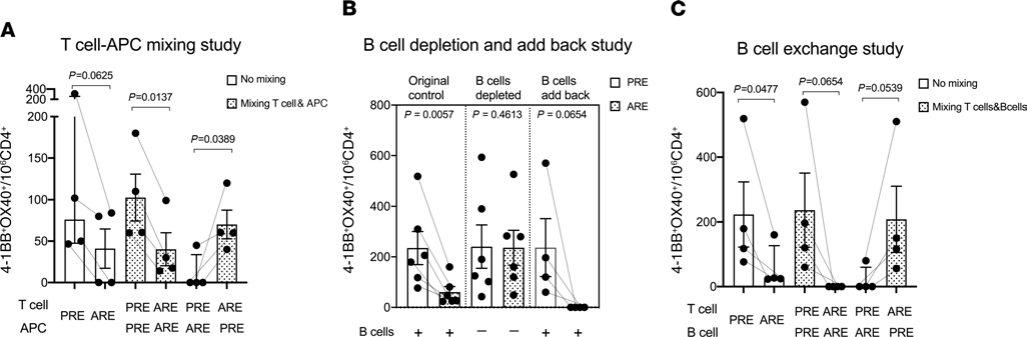
Characterizing the key regulatory cell population. (**A**) T cell–APC mixing study (*n* = 4). T cell responses from pre-break (PRB) samples and after reexposure (ARE) samples without mixing are shown (white) as control. T cells and APCs were separated from samples of both visits and mixed with each other. T cell responses from all 4 mixing combination samples are shown (shaded bar). (**B**) B cell depletion and add-back study. PRE samples are shown in white and ARE samples are represented by shaded bars. T cell responses from original PRE and ARE samples without B cell depletion are shown on the left as controls (*n* = 6). The T cell response of the same 6 samples after B cell depletion are shown in the middle. Four subjects had their B cells depleted first and then added back to the original samples; those T cell responses are shown on the right. (**C**) B cell exchange study (*n* = 4). T cell responses from the original PRE samples and ARE samples are shown in white as control. The shaded bars represent T cell responses from samples with B cells separated first, and then either added back to the original samples (middle) or exchanged between PRE and ARE samples (right). Antigen-specific T cell responses with positive AIM signals (4-1BB^+^OX40^+^) are represented by numbers per million of CD4 T cells. Normality of distribution was accessed by Shapiro-Wilk test. Statistical analysis of nonparametric data was performed by Wilcoxon’s test (1-tailed), and statistical analysis of parametric data was performed by paired Student’s t test (1-tailed). Data are plotted as median with interquartile range for nonparametric data and mean ± SEM for parametric data.

To gain insight in the mechanism of the allergen-specific immune modulation, further mixing studies were performed to identify cell subpopulations associated with suppression of CD4 T cell response. Purified T cells and antigen-presenting cells (APCs) from PBMCs from the PRE and ARE groups were mixed according to their original ratio, and mouse allergen reactive T cell responses were quantified using the AIM assay. Results from 4 different subjects are shown in Figure 3A. As expected, mixing of T cells and APCs both from PBMCs ARE showed similar downregulation of T cell responses as the unseparated samples. Surprisingly, a significant decrease of responses was observed when T cells from PRE samples were mixed with APCs from ARE samples (*P* = 0.0389). Conversely, no suppression was observed in the groups mixing APCs from PRE samples and T cells derived from either ARE or PRE samples. These results imply that it is the APCs from the ARE samples, but not T cells, that mediate the mouse allergen-specific immune modulation.

### B cells are the key modulators among APCs in allergen-specific immune modulation.

To further identify the APC subset(s) involved in the downregulation of T cell responses, we performed APC subset depletion and add-back experiments. We postulated B cells as potential candidates, based on well-known roles of IgE-producing B cells in allergy (16–18), and our phenotyping data, which suggested alterations in the B cell subset. The T cell responses to mouse allergens before and after B cell depletion were measured by the AIM assay. It was noted that, with B cells, depleted suppression of T cell responses in ARE samples was lost, and after B cells were added back, the suppression effect of T cells in ARE samples came back, whereas B cell depletion did not affect T cell responses in the PRE samples (Figure 3B). This indicates that B cells are likely the key APC subset that is responsible for the downregulation of CD4 T cell responses observed ARE.

Further mixing studies, similarly to the APCs mixing experiments above, were performed by exchanging B cells from different time points. Results from 4 different subjects are shown in Figure 3C. As expected, we confirmed the suppressive effect originally observed in all 4 subjects (*P* = 0.0477) and also after adding back B cells from the same visits (*P* = 0.0654). However, by exchanging PRE B cells with ARE B cells, PRE T cell responses were similarly suppressed (*P* = 0.0539), whereas after mixing with PRE B cells, the suppression effect on ARE T cells was lost (Figure 3C). Although not reaching statistical significance due to the limited number of subjects, these results suggest that B cells are the cell type present in the APC preparations responsible for inhibiting T cell responses and highlight that B cells are responsible for the suppression of allergen-specific T cell responses ARE.

### Increased regulatory cytokine production in cell culture supernatant of ARE samples.

**Figure 4:**
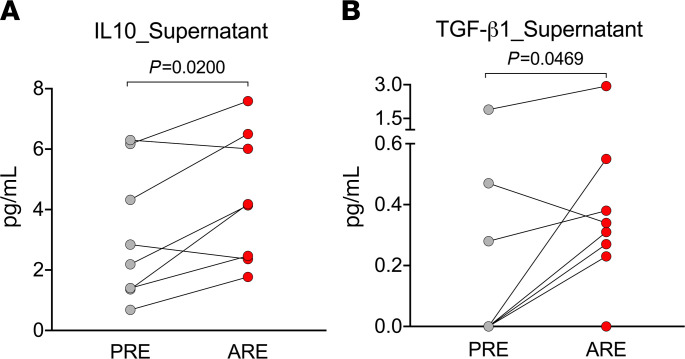
Increased regulatory cytokine production in cell culture supernatant of ARE PBMCs stimulated with mouse allergens. (**A**) IL-10 responses elicited by HiMO in supernatant of a 24-hour stimulation assay in PRE (gray) and ARE (red) PBMC samples (*n* = 8). Six of eight subjects had increased IL-10 production in ARE samples. (**B**) TGF-β1 responses elicited by HiMO in supernatant of a 24-hour stimulation assay in PRE (gray) and ARE (red) PBMC samples (*n* = 8). Six of eight subjects had increased TGF-β1 production in ARE samples. Statistical analysis was performed by Wilcoxon’s test (2-tailed).

Next, a more extensive characterization of the suppressive mechanism in terms of regulatory cytokines was performed. Supernatant of cell culture from 8 subjects that showed suppression effect was collected and tested for IL-10 and TGF-β1 levels after 24-hour stimulation with mouse allergens. As shown in Figure 4, it was found that IL-10 and TGF-β1 levels were increased ARE in ARE samples (*P* = 0.0200 and *P* = 0.0469, respectively). Taken together, these results suggest that B cells might suppress allergen-specific T cell responses through the secretion of regulatory cytokines, such as IL-10 and TGF-β1.

### Increased mouse allergen-specific IgG4 levels ARE.

**Figure 5:**
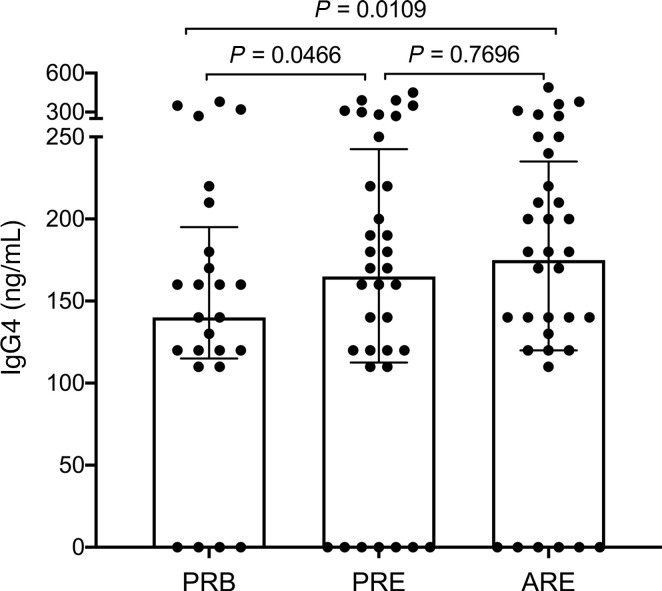
Increased plasma levels of mouse-specific IgG4 observed in the longitudinal study. Plasma levels of mouse-specific IgG4 in laboratory animal-care workers enrolled in the longitudinal study at 3 different visits: PRB, PRE, and ARE (*n* = 36, 11 subjects did not have plasma samples saved at the PRB visit). Data are plotted as mean ± SEM. Statistical analysis was performed by mixed-effects analysis with Tukey’s test corrected for multiple comparisons.

Since IgG4 response in the absence of IgE is suggested to protect against the development of allergic sensitization (19, 20), we sought to measure the modulation of IgG4 plasma levels before and after the reexposure to mice. As shown in Figure 5, a significant increase of plasma IgG4 levels was observed from the ARE (*P* = 0.0109) and PRE (*P* = 0.0466) samples compared with those from PRB samples. Overall, this result suggests that the modulation of mouse-specific responses might involve reciprocal interplay between T cells and B cells.

## Discussion

Earlier studies on immune tolerance highlighted suppression of T cell reactivity as a possible regulatory mechanism in nonallergic individuals (21, 22). More recently, reduced CD4 T cell responses after exposure to allergens has been reported in seasonal pollen allergy (9). Our group previously reported detectable CD4 T cell responses in ENA individuals (14) using developed peptide pools of mouse allergens (12, 13). In the present study, similar downregulation of CD4 T cell responses was observed in mouse allergy among ENA laboratory animal-care workers ARE to mouse allergen, as evidenced by a decrease both in proportion of activated 4-1BB^+^OX40^+^ CD4 T cells and in IFN-γ and IL-5 cytokine production.

Two features of the above immune modulation in response to reexposure to allergens were revealed. First, the immune modulation is highly antigen specific with no bystander effect, as it only suppresses T cell responses to the specific (mouse) allergens, leaving responses to other (PT) antigens intact, even in the presence of the mouse antigens. This ensures targeted off-switch of allergic reactions to specific allergens, while maintaining the functional integrity of the immune system. However, the observations that the inhibitory effect to mouse allergen responses is antigen specific and not expanded to bystander antigens should be validated in future studies using peptide MHC II tetramers as well as with a larger sample size.

Second, decrease in CD4 T cell responses was not reversed by replenishing immune cells from the PRE group (with higher responses before reexposure), suggesting the downregulation of T cell responses is not due to redistribution of immune cells, but an active and dynamic process that protects the ENA subjects from allergic reactions ARE to mouse allergens. While allergy is commonly attributed to pathological immune reactions absent in nonallergic individuals, missing the above protective immune regulation may also be a cause of allergy.

Antigen-specific Tregs have been previously postulated as regulators in the above regulatory process (23). However, the failure of purified T cells from the ARE group to suppress CD4 T cell responses after mixing with APCs from the PRE group hinted that T cells are more likely the targets of this modulation. Furthermore, a transcriptomic study on allergen-specific T cells by Hinz et al. did not yield any significant finding on Treg populations (9), indicating that the mediators may be from other cell types, such as B cells or other APCs (24–27).

We demonstrated that downregulation of CD4 T cell responses in ARE was reversed by depletion of B cells and the suppressive effect was reproduced after B cells were added back, implying that B cells are likely the key regulators. Further B cell exchange study showed that ARE B cells were able to suppress PRE T cells; these results confirmed that B cells are the cell type present in the APCs responsible for the allergen-specific modulation (suppression) ARE. In future studies, a dilution series of B cells in mixing studies with PRE and ARE samples will have to be considered to elucidate the mechanisms and thresholds of suppression. In addition, one important limitation of this study is that our findings do not address how the phenotype of antigen-specific B cells changes after antigen reexposure. It would be certainly interesting to evaluate if they are critically required to mediate the suppressive activity.

Intriguingly, the number of total B cells also decreased ARE. A possible explanation is that the above immune regulation was carried out by a subgroup of B cells that also downregulate the rest of the B cell subpopulations, such as regulatory B cells (Bregs). Bregs have been described as a heterogeneous group of B cells that exert regulatory functions, including IL-10–secreting B cells, marginal zone B cells, TIM-1^+^ B cells, etc. (16). Downregulation of CD4 T cell proliferation and function has been found in multiple subsets of Bregs through mechanisms, including both secretion of antiinflammatory cytokines and direct cell contact (16, 17). Although our study found increased regulatory cytokines (IL-10 and TGF-β1) in cell culture supernatant of PBMC samples ARE, which suggests that a subset of B cells might suppress the allergen-specific responses through secretion of regulatory cytokines, such as IL-10 and TGF-β1, we do not have direct evidence that those regulatory cytokines were produced by B cell subsets. Further studies are needed to investigate in depth the contribution of Bregs in the overall observed phenomenon.

The present study has not fully elucidated how the B cells inhibit immune reactivity to mouse antigens. More studies will address whether cell contact is required and the involvement of neutralizing antibodies in the suppressive activities of B cells. Of note, the IgG4 response in the absence of IgE is suggested to protect against the development of allergic sensitization (19, 20). Therefore, it is reasonable to speculate that the observed modulation of IgG4 ARE to mice could be associated with a protective effect or control of mouse sensitization. It would certainly be of interest to elucidate this hypothesis in ensuing occupational prospective studies.

Understanding the time course of this immune regulation is significant to guide clinical management. Our nonlinear regression model based on cross-sectional data from the first cycle of our longitudinal study implies a phase of progressive downregulation of T cell responses, a trough around 90 days ARE, and a phase of returning toward the baseline. Although a trend of both phases was seen in the second cycle of the longitudinal cohort, the trough was not determined in the present study, which was unfortunately not completed due to the lockdown for the COVID-19 pandemic.

This study is limited by the small sample size of subjects and insufficient longitudinal samples beyond 50 days and in the second cycle ARE. Further details on the time course and mechanisms of the regulatory process of CD4 T cell responses in nonallergic individuals may be elucidated by large-scale longitudinal studies over at least 3–6 months and transcriptomic studies with a focus on B cell subpopulations in the future. In addition, one important limitation of this study is that lymphocyte responses to allergens typically take place in lymph nodes, where the immune cell composition is drastically different than in circulation. It would be very interesting to assess this research question in vivo by the use of fine needle aspirates in future studies.

In summary, using recently developed mouse allergen peptides as tools, we uncovered the antigen-specific immune modulation in a mouse allergy model using samples from laboratory animal-care workers. This study presents potentially novel insights into the active and dynamic nature of the nonallergic status, which involves collaborative interaction between B and T cells. Understanding the mechanisms associated with this allergen-specific immune modulation in healthy nonallergic individuals might be the key to recapitulating immune tolerance and pathophysiology involved in allergy, which could improve the development of diagnostic and therapeutic modalities and bring hope for a cure for allergic diseases in future.

## Methods

### Study design of first-cycle longitudinal study on ENA workers.

Previous work demonstrated that when reexposed to mouse allergens, ENA individuals have lower but still sizable CD4 T cell reactivity as well as absent polarization compared with an allergy group. Moreover, the protein targets of ENA reactivity are similar to those in symptomatic subjects (14). These observations prompt further investigation on additional differences in immune profiles between ENA and allergic individuals and whether the nonallergic status is a passive consequence or active response to mouse allergens.

To address these questions, we longitudinally followed a cohort of vivarium and research laboratory ENA subjects working in contact with mice. In addition, based on previous observations in a seasonal pollen allergy study (9), we introduced breaks of exposure to evaluate immune modulation after break and reexposure to mouse allergens. Peripheral blood samples were collected from subjects continuously exposed (PRB), after break but before reexposure (PRE), and ARE (Figure 1A).

### Study design of second-cycle longitudinal study.

To further explore the dynamic change of T cell modulation ARE suggested by the nonlinear regression model based on cross-sectional data from the first cycle of our longitudinal study, subjects who participated in the first cycle of the longitudinal study were recalled, and a second cycle of longitudinal study was subsequently designed with 5 follow-up visits: PRB, PRE, ARE1, ARE2, and ARE3. Unfortunately, due to the lockdown for the global COVID-19 pandemic, only 4 subjects completed the ARE1 visit in the second cycle (Supplemental Figure 2, A–D).

### Study cohort and PBMC isolation.

The study cohort recruited for this study included 36 healthy laboratory animal-care workers from San Diego, California, USA. Each participant was assigned a study identification number with clinical information recorded. Clinical symptoms of allergy were collected by questionnaire-based survey and IgE titers were determined from plasma using the Phadia’s ImmunoCAP assay (Thermo Fisher Scientific).

PBMCs were isolated from whole blood by density gradient centrifugation according to the manufacturer’s instructions (Ficoll-Hypaque, Amersham Biosciences) and cryopreserved for further analysis.

### Peptide synthesis and megapool generation.

We synthesized a total of 106 epitopes previously identified from the high-molecular-weight fraction of mouse allergen extracts (12). Peptides were derived from (a) Mus m 1, (b) Mus m 1-isoforms, (c) mouse proteins homologous to major mammalian allergens, and (d) peptides identified by immunoproteomic analysis of the high-molecular-weight fractions of mouse allergen extracts and conventional allergens (herein referred to as HiMO peptides; ref. 12). Peptides were purchased from A and A as crude material on a small (1 mg) scale. Individual peptides were resuspended in DMSO at a final concentration of 40 mg/ml. The selected peptides were pooled together and underwent sequential lyophilization, as described previously (15), and were then resuspended in DMSO to a final concentration of 2 mg/ml. The production of control PT peptides and megapool were previously described (28). Experimentally validated peptides were selected from a total of 785 peptides tested from the *Bordetella pertussis* strain Tohama I, and individual peptides were synthesized by A and A and resuspended to a final concentration of 1 mg/mL in DMSO.

### Dual ELISPOT cytokine assay.

IFN-γ and IL-5 secretion from stimulated PBMCs was measured by dual ELISPOT assays as previously described (29). Briefly, cells (1 × 10^5^ cells/well) were stimulated in triplicate with HiMO pool (5 μg/ml), PHA (10 μg/ml) as a positive control, or medium containing 0.25% DMSO (percentage of DMSO in the pools/peptides) as a negative control. Spot-forming cells (SFCs) were counted by computer-assisted image analysis (AID iSpot EliSpot/FluoroSpot reader, Autoimmun Diagnostika GmbH). Positivity was defined as ≥100 SFCs for peptide pools per 10^6^ PBMCs, *P* < 0.05 based on Poisson’s test between negative control replicates and stimuli replicates, and a stimulation index (SI) ≥2 (SI is calculated as the average SFCs for stimuli divided by the average SFCs for negative control). Positive peptide pools were deconvoluted to identify the individual response.

### AIM assay for CD4 T cell responses.

The AIM_OX40/CD137_ assay was used to detect T cells activated due to allergen-specific stimulation by upregulation of activation-induced surface markers (30). Briefly, frozen PBMCs were thawed and stimulated in 5% human serum (Gemini Bioproducts) for 24 hours by HiMO megapool (2 μg/ml), DMSO (0.25%) as negative control or PHA (1 μg/ml) as positive control. Alternatively, responses against a pool of peptides derived from *B*. *pertussis* (PT) were used (15). After incubation, cells were stained with a cocktail containing the antibodies described in (Supplemental Table 1). Stained cells were acquired in a BD LSRII Flow Cytometer and analyzed by FlowJo X Software (version 10, Tree Star).

### Ex vivo phenotypic characterization.

Phenotypic characterization of PBMC subset populations directly ex vivo and without antigen stimulation was performed by direct staining with antibody cocktails at 4°C for 30 minutes for extracellular staining. Populations that were assessed, flow cytometry antibodies, and gating strategy are listed in Supplemental Table 2. For assessment of Tregs, T follicular helper cells, and follicular Tregs subsets, following extracellular staining, cells were washed, fixed with 4% paraformaldehyde, permeabilized with 0.5% saponin, and stained for intracellular markers (Supplemental Table 1). Cells were acquired in a BD LSRII Flow Cytometer and analyzed by FlowJo X Software (version 10, Tree Star).

### Before reexposure and ARE cell mixing study.

Coculture of longitudinal samples (PRE and ARE mixed by 1:1 ratio) stimulated by mouse (HiMO) and PT antigens was performed to test the dominance of the suppression effect observed in mouse allergy. Eight subjects with downmodulation of T cell responses in ARE samples observed in previous assays were selected, and their PRE and ARE PBMC samples were mixed in a 1:1 ratio and stimulated by mouse allergen (HiMO) for 24 hours. The T cell responses of PRE, ARE, and mixed cultures were detected by AIM assay with surface markers of 4-1BB and OX40. PRE and ARE cell cultures were also stimulated with PT alone and PT mixed with mouse allergens simultaneously to test the antigen specificity of the suppression effect observed, T cell responses of PT alone, and PT + HiMO were detected by AIM assay for analysis.

### T cell–APC mixing study.

CD3^+^ T cells were purified using the human T cell negative selection kit (EasySep Human T cell Isolation Kit, Stemcell Technologies). APCs were separated from PBMCs in the negative flow through using the human CD3 positive selection kit (EasySep Human CD3 Positive Selection Kit II, Stemcell Technologies). We conducted experiments by selecting 4 subjects whose responses were observed to be downmodulated in previous assays; their purified T cells and APCs from PBMCs before reexposure and ARE were mixed according to the original ratio, and mouse allergen-reactive T cell responses were detected using AIM assay.

### B cell depletion and add-back and exchange study.

We conducted experiments by selecting 6 subjects whose responses were observed to be downmodulated in previous assays, and the B cells in both PRE and ARE samples were separated using the human CD19 positive selection kit (EasySep Human CD19 Positive Selection Kit II, Stemcell Technologies). The mouse allergen-specific T cell responses in cultures encompassing from B cell–depleted APCs were measured using the AIM assay. For 4 of the subjects, the B cells were added back to the original samples or exchanged between PRE and ARE samples, and their mouse-specific T cell responses were measured again using the same AIM assay.

### Regulatory cytokine (IL-10 and TGF-β1) production.

PBMCs from 8 subjects that shown suppression effect were stimulated with mouse allergens for 24 hours, and their cell culture supernatant was collected and stored at –80°C until subsequent analyses. IL-10 and TGF-β1 levels were measured using the LEGENDplex Human B cell Panel (13-plex; BioLegend) and Human TGF-β1 assay (1-plex, Biolegend) according to the manufacturer’s recommendations and analyzed using the BD LSRII Flow Cytometer and the BioLegend LEGENDplex software.

### Radioallergosorbent test.

Plasma mouse allergen-specific IgE and IgG4 levels were measured by the radioallergosorbent test (RAST) with ImmunoCap assay (E88) (Thermo Fisher Scientific) performed at Phadia immunology reference laboratory.

### Statistics.

Experimental data were analyzed by GraphPad Prism (version 8), Microsoft Excel, and R (version 3.3.3). Statistical details of the experiments are provided in the respective figure legends. Data are plotted as median with interquartile range for nonparametric data and mean ± SEM for parametric data. Normality of distribution was accessed by Shapiro-Wilk test. Nonparametric data were compared by Wilcoxon’s test for paired comparison, Friedman’s test corrected for multiple comparisons using Dunn-Bonferroni test, and Spearman’s correlation analysis. Parametric data from the longitudinal study were compared by paired Student’s *t* test and mixed-effects analysis with Tukey’s test corrected for multiple comparisons (for comparing groups with missing values). All the statistical comparisons were performed as 2 tailed unless specified otherwise in the figure legends. *P* values of less than 0.05 were defined as statistically significant. Nonlinear generalized additive model was performed using the mgcv package for R.

### Study approval.

This study was approved by the Institutional Review Board of La Jolla Institute for Immunology (IRB protocol no. VD-145). Each participant provided informed consent and was assigned a study identification number with clinical information recorded.

## Author contributions

EDY, RDSA, and A. Sette designed research studies. EDY, AG, LW, A. Sutherland, EW, and RDSA provided investigation. EDY, RDSA, and A. Sette analyzed data. AF, RDSA, and A. Sette provided resources. EDY, RDSA, and A. Sette wrote the manuscript. BP, RDSA, and A. Sette supervised the studies. AF provided project administration. RDSA and A. Sette acquired funding.

## Supplementary Material

Supplemental data

## Figures and Tables

**Table 1:**
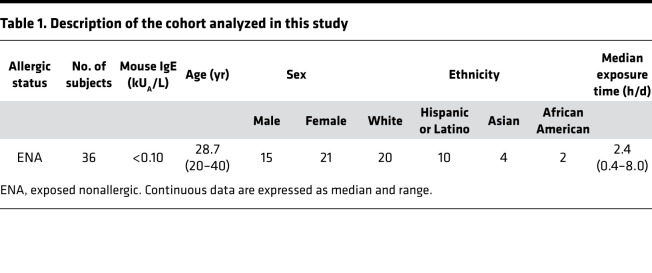
Description of the cohort analyzed in this study

## Version 1 02/22/2021

Electronic publication
