# Supplementary material for: B cells modulate mouse allergen-specific T cells in nonallergic laboratory animal-care workers
Source: JCI Insight. 2021 Feb 22;6(4):e145199. doi: 10.1172/jci.insight.145199 (PMC7934936; doi:10.1172/jci.insight.145199)
Supplement: Supplemental data [file jciinsight-6-145199-s224.pdf]

Supplemental Materials for

B cells modulate mouse allergen-specific T cells in non-allergic laboratory animal-care workers

Esther Dawen Yu[1], Luise Westernberg[1], Alba Grifoni[1], April Frazier[1], Aaron Sutherland[1], Eric Wang[1], Bjoern Peters[1,2], Ricardo da Silva Antunes[1,3,*] and Alessandro Sette[1,2,3,*]

[1]La Jolla Institute for Immunology, La Jolla, California, USA

[2]Department of Medicine, University of California San Diego, La Jolla, California, USA

[3] These authors contributed equally

Conflict of interest statement: The authors have declared that no conflict of interest exists.

[*]Correspondence should be addressed to Ricardo da Silva Antunes, La Jolla Institute for Immunology, La Jolla, CA 92037, USA, phone: +1-858-752-6500, email: rantunes@lji.org and Alessandro Sette, La Jolla Institute for Immunology, La Jolla, CA 92037, USA, phone: +1-858-752-6919, email: alex@lji.org.

Supplemental Figure 1

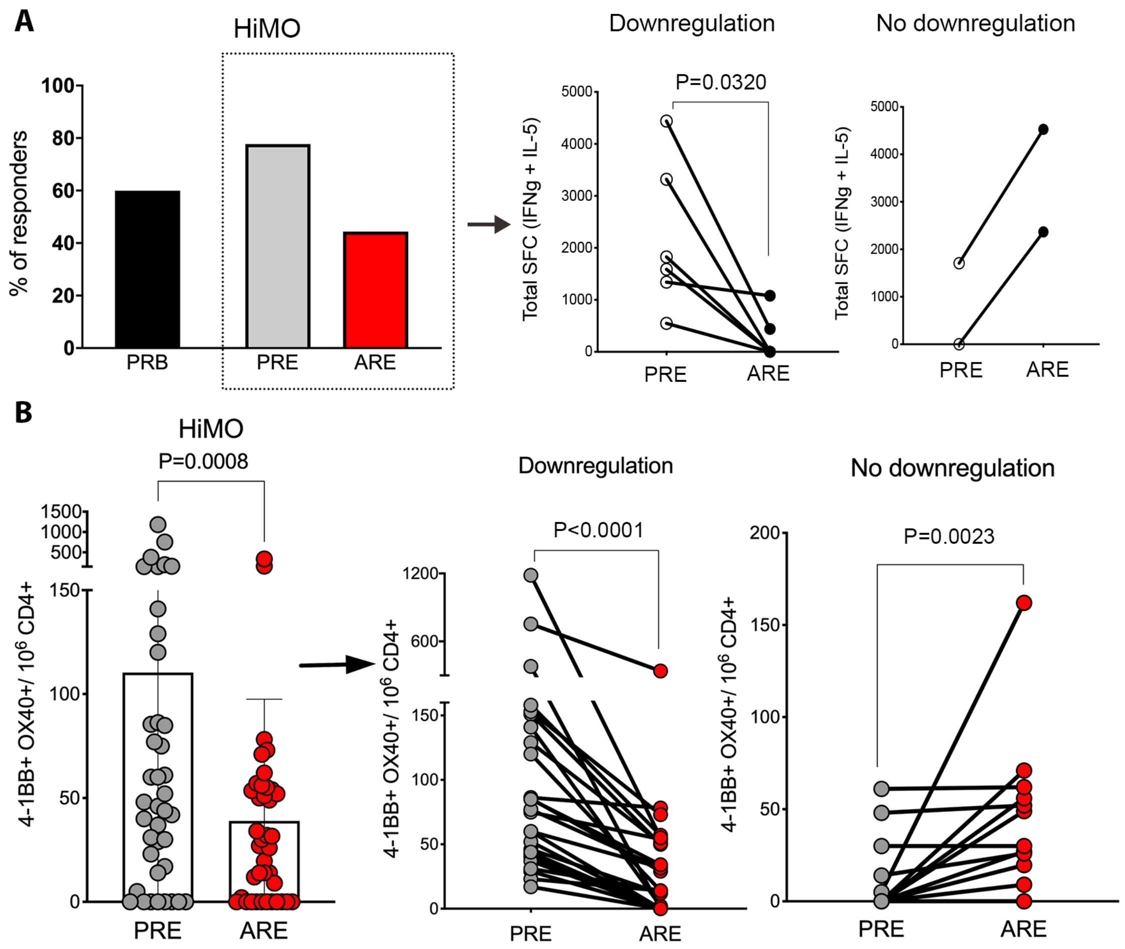

Supplemental Figure 1: Down regulation of cytokine secretion and T cell responses in exposed non-allergic (ENA) subjects upon re-exposure to mouse allergens.

(**A**) Cytokine responses (IFNγ, IL-5) elicited by HiMO in a 14-day in vitro restimulation assay in PRB, PRE, and ARE groups were shown (n =8). (**A**, right panel) 6 out of 8 subjects showed down regulation of cytokine secretion after re-exposure and 2 of 8 subjects did not show down regulation. (**B**) Activated T cell reactivities were detected using AIM assay (n = 36), AIM positive signals (4-1BB^{+} OX40^{+}) were represented by numbers per million of CD4 T cells. Activated T cell responses in the ARE group was highlighted in red. (**B**, right panel) 25 out of 36 subjects showed down regulation of T cell responses after re-exposure and 11 of 36 subjects

did not show down regulation of T cell responses. Data were plotted as median with interquartile range. Statistical analysis was performed by Wilcoxon test (two-tailed).

Supplemental Figure 2

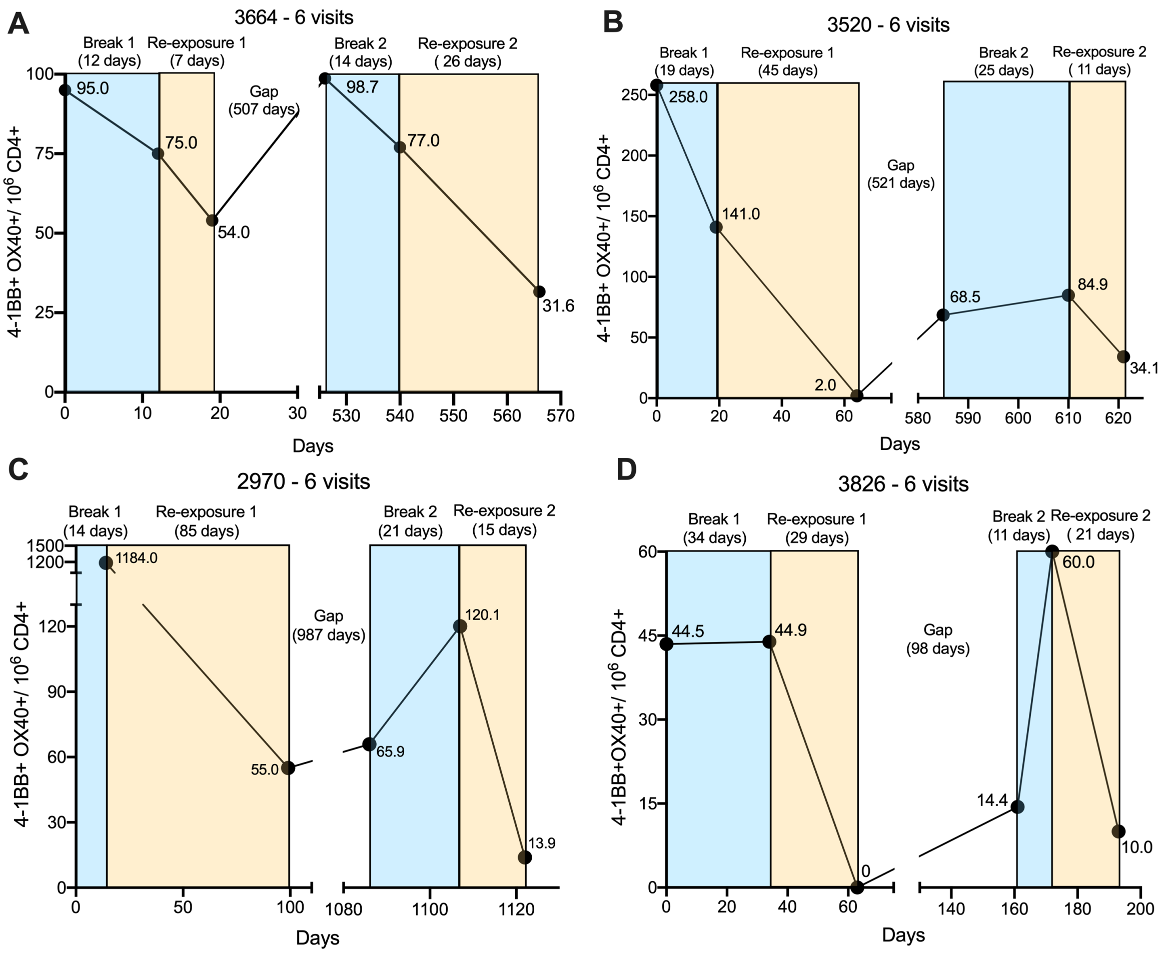

Supplemental Figure 2: Time course of CD4 T cell responses over 2 cycles of break and re-exposure from mouse antigens.

(**A-D**): Four subjects were followed for two break and re-exposure cycles with 6 longitudinal visits for better illustration of the cyclic response fluctuation of T cell responses after each break and re-exposure to mouse. Break period was illustrated in blue, re-exposure time was in yellow, and gap was left blank, exact duration of each was indicated in days. T cell responses were detected by AIM assay, AIM positive signals (4-1BB^{+} OX40^{+}) were represented by numbers per million of CD4 T cells.

Supplemental Figure 3

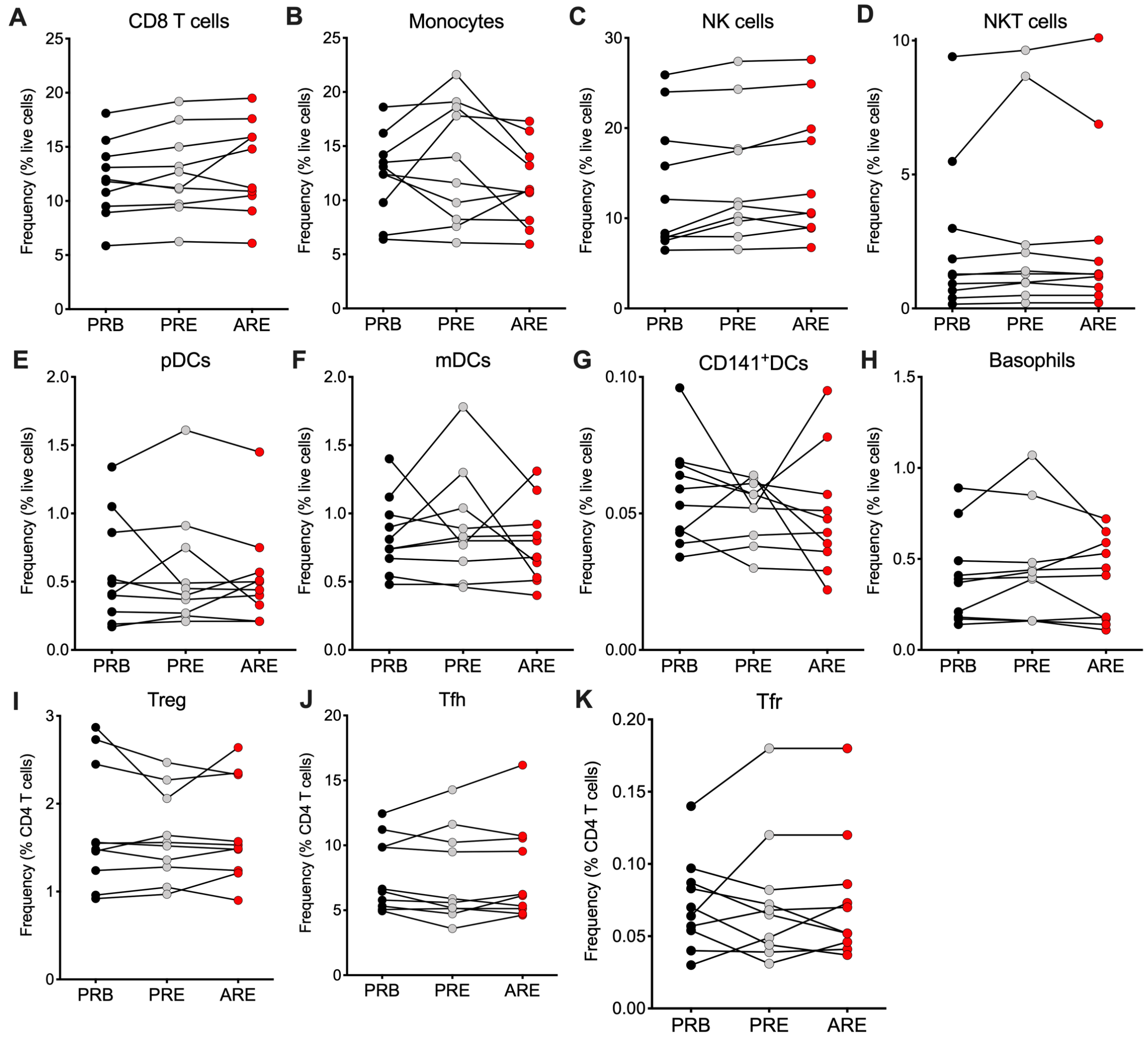

Supplemental Figure 3: Immunophenotyping of other PBMC subsets from Pre/Post re-exposure samples.

Ex vivo immunophenotyping of other PBMC subsets did not reveal significant changes from Pre/Post re-exposure samples (n=10), data were summarized in (**A**) CD8 T cells, (**B**) monocytes, (**C**) natural killer cells (NK cells), (**D**) natural killer T cells (NKT cells), (**E**) plasmacytoid dendritic cells (pDCs), (**F**) myeloid dendritic cells (mDCs), (**G**) CD141^{+} dendritic cells (CD141^{+}DCs), (**H**) basophils, (**I**) T regulatory cells (Treg), (**J**) T follicular helper cells (Tfh), and (K) T follicular regulatory cells (Tfr). Statistical analysis was performed by Friedman test corrected for multiple comparisons using Dunn-Bonferroni test.

Supplemental Figure 4

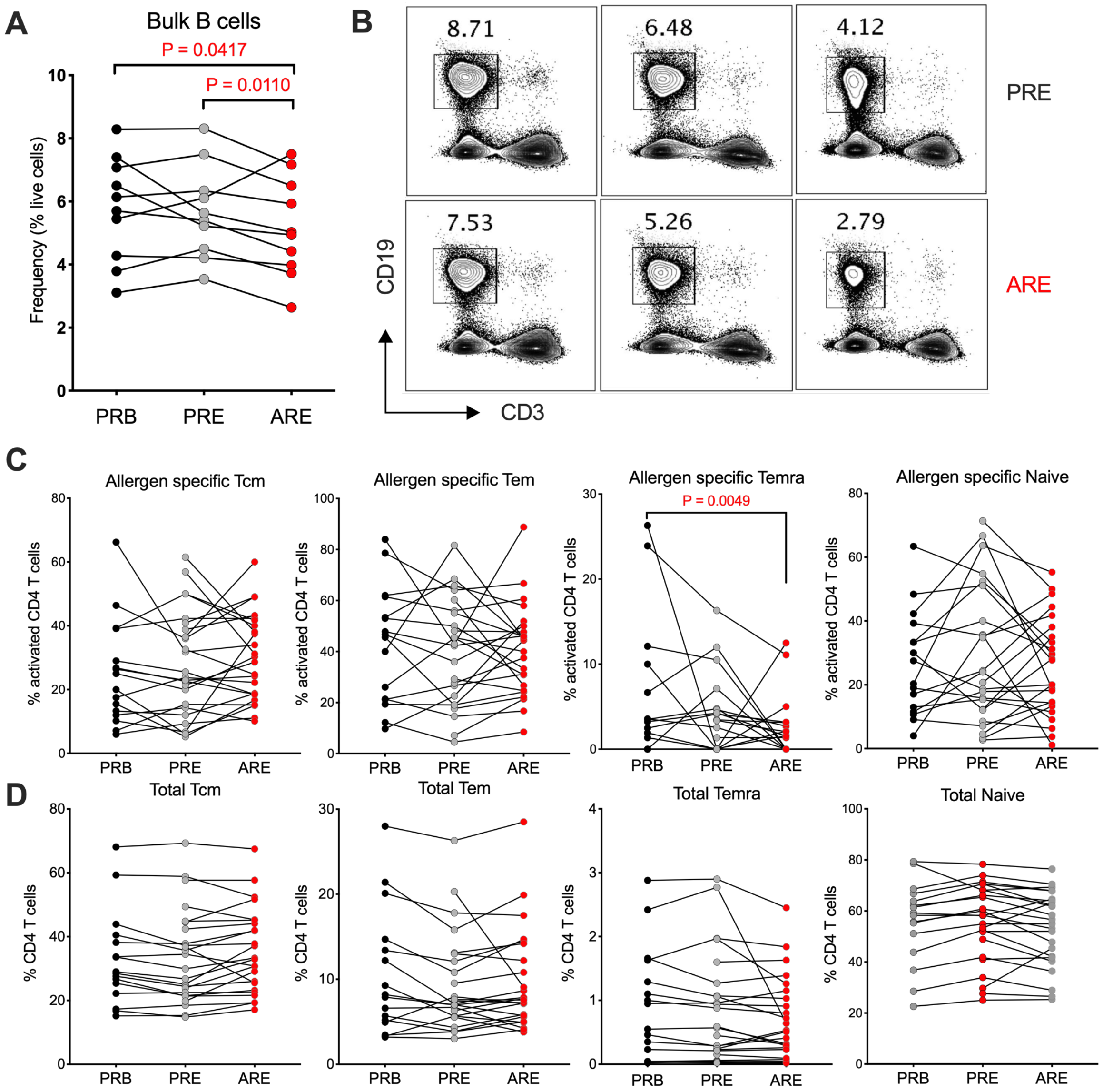

Supplemental Figure 4: Immunophenotyping of PBMC revealed changes in bulk B cells and antigen specific Temra cells.

(**A**) Ex vivo Immunophenotype of PBMC in non-allergic subjects with down modulation of T cell responses (n = 10) revealed that bulk B cells ($CD14^{-}CD3^{-}CD19^{+}$) from 9 out of 10 subjects were also downregulated. Statistical analysis was performed by Friedman test corrected for multiple comparisons using Dunn-Bonferroni test. B cells responses with representative FACS plots were shown in (**B**). Comparison of total and mouse specific memory cells and their subtypes between three groups (PRB, PRE and ARE) were summarized in (**C and D**). Total 24

subjects included in the analysis, and 8 subjects did not have PRB samples left for testing. 9 out of 16 subjects with PRB samples had decreased mouse specific Temra ($CD45RA^{+}CCR7^{-}$) cells at ARE visit. Statistical analysis was performed by Mixed-effects analysis with Tukey test corrected for multiple comparisons.

Supplemental Table 1. List of antibodies used in the activation induced markers (AIM) assay and ex vivo immunophenotyping

Antibody	Fluorochrome	Clone	Vendor	staining	Type of assay
CD3	AF700	UCHT1	BD Biosciences	extracellular	$AIM_{OX40/CD137}$/ex vivo
CD4	APCef780	RPA-T4	eBiosciences	extracellular	$AIM_{OX40/CD137}$/ex vivo
CD8a	V500	RPA-T8	BD Biosciences	extracellular	$AIM_{OX40/CD137}$
CD14	V500	M5E2	BD Biosciences	extracellular	$AIM_{OX40/CD137}$
CD19	V500	HIB19	BD Biosciences	extracellular	$AIM_{OX40/CD137}$
CD45RA	eF450	HI100	eBiosciences	extracellular	$AIM_{OX40/CD137}$/ex vivo
CCR7	PerCP/Cy5.5	G043H7	Biolegend	extracellular	$AIM_{OX40/CD137}$/ex vivo
OX40	PE-Cy7	ACT35	Biolegend	extracellular	$AIM_{OX40/CD137}$
CD137	APC	4B4-1	Biolegend	extracellular/intracellular	$AIM_{OX40/CD137}$
Live/Dead Viability	eF506	-	eBiosciences	intracellular	$AIM_{OX40/CD137}$/ex vivo
CD8a	BV650	RPA-T8	Biolegend	extracellular	Ex vivo
CD19	PECy7	HIB19	TONBO	extracellular	Ex vivo
CD14	APC	61D3	TONBO	extracellular	Ex vivo
CD56	PE	CMSSB	eBiosciences	extracellular	Ex vivo
CD25	FITC	M-A251	BD Biosciences	extracellular	Ex vivo
CD11c	AF780	N418	eBiosciences	extracellular	Ex vivo
CD1c	AF700	L161	Biolegend	extracellular	Ex vivo
CD123	BV650	6H6	Biolegend	extracellular	Ex vivo
CD141	PECy7	M80	Biolegend	extracellular	Ex vivo
HLA-DR	PE	L243	Biolegend	extracellular	Ex vivo
Lin 2	FITC	UCHT1, HCD14, HIB19, 2H7, HCD56	Biolegend	extracellular	Ex vivo
CD16	eF450	CB16	eBiosciences	extracellular	Ex vivo
CD45RO	FITC	UCHL1	Biolegend	extracellular	Ex vivo
CXCR5	BV421	RF8B2	BD Biosciences	extracellular	Ex vivo
CD25	BV605	2A3	BD Biosciences	extracellular	Ex vivo
CD127	PECy7	HIL-7R-M21	BD Biosciences	extracellular	Ex vivo
Fox P 3	PE	206D	Biolegend	intracellular	Ex vivo

Supplemental Table 2. Identification of immune cell subsets in human PBMCs by flow cytometry

Population name	Gating Strategy	Type of assay
CD4+ T cells	CD8-CD14-CD19-CD3+CD4+OX40+CD137+	$AIM_{OX40/CD137}$
CD4+ naive	CD8-CD14-CD19-CD3+CD4+OX40+CD137+CD45RA+CCR7+	$AIM_{OX40/CD137}$
CD4+ Tcm	CD8-CD14-CD19-CD3+CD4+OX40+CD137+CD45RA-CCR7+	$AIM_{OX40/CD137}$
CD4+ Tem	CD8-CD14-CD19-CD3+CD4+OX40+CD137+CD45RA-CCR7	$AIM_{OX40/CD137}$
CD4+ Temra	CD8-CD14-CD19-CD3+CD4+OX40+CD137+CD45RA+CCR7-	$AIM_{OX40/CD137}$
Monocytes	CD14+	Ex vivo staining (Panel 1)
B cells	CD14-CD3-CD19+	Ex vivo staining (Panel 1)
CD3+ T cells	CD14-CD3+CD19-CD56-	Ex vivo staining (Panel 1)
NKT cells	CD14-CD3+CD19-CD56+	Ex vivo staining (Panel 1)
NK cells	CD14-CD3-CD19-CD56+	Ex vivo staining (Panel 1)
CD4+ T cells	CD14-CD3+CD19-CD56-CD4+CD8-	Ex vivo staining (Panel 1)
CD8+ T cells	CD14-CD3+CD19-CD56-CD4-CD8+	Ex vivo staining (Panel 1)
CD4+ naive	CD14-CD3+CD19-CD56-CD8-CD4+CD45RA+CCR7+	Ex vivo staining (Panel 1)
CD4+ Tcm	CD14-CD3+CD19-CD56-CD8-CD4+CD45RA-CCR7+	Ex vivo staining (Panel 1)
CD4+ Tem	CD14-CD3+CD19-CD56-CD8-CD4+CD45RA-CCR7-	Ex vivo staining (Panel 1)
CD4+ Temra	CD14-CD3+CD19-CD56-CD8-CD4+CD45RA+CCR7-	Ex vivo staining (Panel 1)
CD8+ naive	CD14-CD3+CD19-CD56-CD8+CD4-CD45RA+CCR7+	Ex vivo staining (Panel 1)
CD8+ Tcm	CD14-CD3+CD19-CD56-CD8+CD4-CD45RA-CCR7+	Ex vivo staining (Panel 1)
CD8+ Tem	CD14-CD3+CD19-CD56-CD8+CD4-CD45RA-CCR7-	Ex vivo staining (Panel 1)
CD8+ Temra	CD14-CD3+CD19-CD56-CD8+CD4-CD45RA+CCR7-	Ex vivo staining (Panel 1)
pDCs	CD14-CD3-CD19-CD56-CD16-HLA-DR+CD11c+CD1c-CD123+	Ex vivo staining (Panel 2)
mDCs	CD14-CD3-CD19-CD56-CD16-HLA-DR+CD11c+CD1c+CD123-	Ex vivo staining (Panel 2)
CD141+DCs	CD14-CD3-CD19-CD56-CD16-HLA-DR+CD141+	Ex vivo staining (Panel 2)
Basophils	CD14-CD3-CD19-CD56-CD16-HLA-DR-CD123+	Ex vivo staining (Panel 2)
Treg (Fox P3)	CD8-CD14-CD19-CD3+CD4+CXCR5-CD45RO+CD25+CD127lowFox P3+	Ex vivo staining (Panel 3)
Tfh cells	CD8-CD14-CD19-CD3+CD4+CXCR5+CD45RO+	Ex vivo staining (Panel 3)
Tfr cells	CD8-CD14-CD19-CD3+CD4+CXCR5+CD45RO+CD25+CD127lowFox P3+	Ex vivo staining (Panel 3)

Abbreviations: Tcm, central memory T cells; Tem, effector memory T cells; Temra, terminally differentiated effector memory T cells; NK cells, natural killer cells; NKT cells, natural killer T cells; pDCs, plasmacytoid dendritic cells; mDCs, myeloid dendritic cells; $CD141^+$DCs, $CD141^+$ dendritic cells; Treg, T regulatory cells; Tfh, follicular helper memory T cells; Tfr, T follicular regulatory cells.
